# Acute Exercise-Induced Oxidative Stress Does Not Affect Immediate or Delayed Precursor Cell Mobilization in Healthy Young Males

**DOI:** 10.3389/fphys.2020.577540

**Published:** 2020-10-20

**Authors:** Michelle Schmid, Hans-Jürgen Gruber, Julia M. Kröpfl, Christina M. Spengler

**Affiliations:** ^1^Exercise Physiology Lab, Institute of Human Movement Sciences and Sport, ETH Zurich, Zurich, Switzerland; ^2^Clinical Institute of Medical and Chemical Laboratory Diagnostics, Medical University of Graz, Graz, Austria; ^3^Zurich Center for Integrative Human Physiology (ZIHP), University of Zurich, Zurich, Switzerland

**Keywords:** stem cell mobilization, hematopoietic, endothelial, mesenchymal, apoptosis, total oxidative capacity, oxidative stress index, acute exhaustive exercise

## Abstract

Exercise is known to acutely and transiently mobilize precursor cells to the peripheral blood. To date, the underlying mechanisms have not yet been fully elucidated and we hypothesized that exercise-induced oxidative stress could be a mobilizing agent, either directly or *via* circulating apoptotic cells as mediators. The aim of the study was to assess the effect of acute exercise-induced oxidative stress on numbers of circulating angiogenic precursor cells (CACs), circulating non-angiogenic precursor cells (nCACs), mesenchymal precursor cells (MPCs), mature endothelial cells (ECs), and mononuclear cells (MNCs), as well as their apoptotic subsets. Healthy, young males (*n* = 18, age: 24.2 ± 3.5 years) completed two identical, standardized incremental cycling tests. The first, un-supplemented control test was followed by a 7-day-long supplementation of vitamin C (1,000 mg/day) and E (400 I.U./day), immediately preceding the second test. Blood samples were collected before, directly after, 30, 90, 180, and 270 min after exercise, and aforementioned circulating cell numbers were determined by flow cytometry and a hematology analyzer. Additionally, total oxidative capacity (TOC) and total antioxidative capacity (TAC) were measured in serum at all timepoints. Antioxidative supplementation abolished the exercise-induced increase in the oxidative stress index (TOC/TAC), and reduced baseline concentrations of TOC and TOC/TAC. However, it did not have any effect on CACs, nCACs, and MPC numbers or the increase in apoptotic MNCs following exercise. Our results indicate that exercise-induced oxidative stress is neither a main driver of lymphocyte and monocyte apoptosis, nor one of the mechanisms involved in the immediate or delayed mobilization of precursor cells.

## Clinical Trial Registration

http://clinicaltrials.gov, NCT03747913. Registered 20 November, 2018. Available at: https://clinicaltrials.gov/ct2/show/NCT03747913?term=stem+cell+mobilization%2C+oxidative+stress&cntry=CH&city=Zürich&draw=2&rank=1.

## Introduction

Several studies have shown that an acute stimulus of maximal exercise can elicit a transitory increase in endothelial precursor cells (EPCs; Koutroumpi et al., 2012; Silva et al., 2012; De Biase et al., 2013; Boppart et al., 2015) as well as mature endothelial cells (ECs; Shaffer et al., 2006; Niemiro et al., 2017), hematopoietic precursor cells (HPCs; De Lisio and Parise, 2013; Boppart et al., 2015), and mesenchymal precursor cells (MPCs; Ramírez et al., 2006; Boppart et al., 2015) present in the peripheral blood. Increased numbers of circulating stem and progenitor cells have been implied to have a positive effect on the prognosis and health outcomes of diseases, such as cardiovascular and cardiopulmonary conditions or the metabolic syndrome (Choi et al., 2010; Wojakowski et al., 2012; Rattazzi et al., 2013). Thus, identifying the factors responsible for this exercise-induced rise in circulating stem and progenitor cell numbers may contribute to a better understanding of the known health benefits of exercise in general and may help to better tailor training regimens.

Several mechanisms, such as activation of the sympathetic nervous system, increased shear stress, or a shift in the redox balance have already been proposed in literature (Moebius-Winkler et al., 2011; Kröpfl et al., 2014; Emmons et al., 2016; Kröpfl et al., 2020) but a thorough investigation of the possible players at hand, how they interact and to which extent each of them contributes to the final mobilization has long been lacking. Agha et al. (2018) found the increase in CD34^+^ HPC numbers after vigorous cycling to be reduced upon blockage of *β*_1_- and *β*_2_-adrenergic receptors, which further solidifies the role of sympathetic stress in exercise-induced stem cell mobilization. It was reported; however, that mobilization was indeed reduced but not completely suppressed, suggesting sympathetic stress contributes to, but does not completely account for the rise in circulating stem cell numbers after physical activity. Montgomery et al. (2019) investigated changes in EPC numbers after knee extension exercise with and without blood flow restriction in order to test a possible involvement of shear stress. Contrary to their initial hypothesis of increased shear stress correlating with increased mobilization, they found blood flow restriction to even reduce the exercise-induced increase in circulating EPCs.

It therefore remains to be established if and to which extent exercise-induced oxidative stress plays a role in this process, as reactive oxygen species (ROS) have previously been found to not only act as harmful, cell-damaging agents but also play an important role in the regulation and control of cell signaling pathways and gene expression (Droge, 2002; Powers et al., 2010; Hervera et al., 2019). It has already been shown that some other health-promoting effects of physical activity depend on a transient increase in oxidative stress and that a reduction of this oxidative stress *via* administration of antioxidative supplements can prevent long-term adaptions, such as increases in insulin sensitivity (Ristow et al., 2009). The concept of an acute bout of exercise stimulating the enhanced production of ROS is not new (Davies et al., 1982; Ashton et al., 1998; Miyazaki et al., 2001; Finaud et al., 2006). An increased O_2_ consumption and a subsequent enhanced flux of oxygen through the mitochondria have long been believed to be the primary source of ROS-production during prolonged or high-intensity exercise, until advances in the field pinpointed the key role in exercise-related ROS production to nicotinamide adenine dinucleotide phosphate oxidase in contracting muscles (Sakellariou et al., 2013; Powers et al., 2016). At rest, free radicals are produced at a low rate and are effectively scavenged by the antioxidative defense system. If created at higher rates – such as during exercise – the amount of free ROS may exceed the capacity of the antioxidative system which would produce oxidative stress. Thus, oxidative stress in the blood can be measured by building the ratio of the total oxidative capacity (TOC) to the total antioxidative capacity (TAC). It is known that ROS can oxidize lipids (Morel et al., 1983), proteins (Szweda et al., 2002), and DNA (Wallace, 2002), and thus induce loss of cell viability by driving cells into apoptosis. Interestingly, Mooren and Krüger (2015) investigated the effect of different dosages of apoptotic cells injected into sedentary mice and found a dose-dependent increase in circulating Sca-1^+^/c-kit^+^ progenitor cells 3 h after injection. Building upon this finding, there could be a possible link between the degree of oxidative stress-induced cell apoptosis by physical exercise and the extent of stem and progenitor cells mobilized to the circulation.

The aim of the present study was to compare the oxidative stress index, defined as the ratio of TOC/TAC, the extent of circulating apoptotic mononuclear cells (MNCs), the kinetics of EPCs and HPCs – named circulating angiogenic precursor cells (CACs), circulating non-angiogenic precursor cells (nCACs), MPCs, as well as mature ECs up to 270 min after an acute bout of incremental cycling with and without preceding antioxidative supplementation.

We hypothesized that (1) acute exercise-induced oxidative stress is blunted upon supplementation of antioxidative vitamins C and E, (2) TOC/TAC is related to the number of circulating apoptotic MNCs, and (3) the number of circulating apoptotic MNCs is associated with the extent of the exercise-induced mobilization of CACs, nCACs, and/or MPCs – either directly or with a delayed effect.

## Materials and Methods

### Participants

Eighteen healthy, non-smoking, physically active (at least 150 min of moderate-intensity aerobic physical activity/week or at least 75 min of vigorous-intensity aerobic physical activity/week or any equivalent combination thereof), young males (age 18–35 years) with a body mass index (BMI) between 18.5 and 25 kg·m^−2^ volunteered to participate in the study. Subjects’ characteristics and anthropometrics are shown in Table 1. Presence of any of the following criteria led to exclusion of the participant: known or suspected non-compliance, inability to follow the procedures of the study, previous enrolment into the current study or participation in another study including exercise interventions and/or antioxidant supplementation, acute or chronic conditions and/or regular intake of medication affecting sleep or the performance of the respiratory, cardiovascular, or neuromuscular system, contraindications to acidum ascorbicum (vitamin C), d-alpha-tocopherol (vitamin E), and/or any of the pharmaceutical additives of the provided supplements, regular intake of antioxidative supplementation other than the ones provided during the study period, working with pesticides or metal on a daily basis or intention to do so during the study period, donation of blood within the last 12 weeks or intention to do so during the study period, and dental treatments in the last 3 days before enrollment or intention to undergo any during the study period, newly acquired piercings, tattoos, and permanent make-ups within the last 16 weeks before enrollment or intention to acquire any during the study period. Additionally, over the whole duration of the study, participants were asked to sleep for a minimum of 7 h in the two nights preceding a visit and to refrain from solarium-usage and/or extensive sun exposure without protection throughout the study duration, any engagement in intense exercise within 48 h, and in any exercise within 24 h prior to a visit, any alcohol intake within 48 h before a visit, as well as the intake of caffeine on testing days prior to testing. Furthermore, on the first visit to the laboratory, participants received a list of antioxidative-rich foods and drinks of which the intake was to be held constant over the whole study duration and prohibited on a testing day. Compliance and adherence to the study rules were monitored *via* daily questionnaires on each testing day and with a wrist-worn actigraph (Actiwatch Score, Cambridge Neurotechnology, Cambridge, United Kingdom) tracking sleep and activity over the whole study duration.

**Table 1:** Participant characteristics (*n* = 18).

Age (years)	24.2	±	3.5
Height (m)	1.83	±	0.08
Weight (kg)	77.5	±	9.2
BMI (kg·m^−2^) Body fat (%) Fat mass (kg) Lean mass (kg) Bone mass (kg) Bone density (g·cm^−1^)	23.1 15.3 11.51 63.42 3.40 1.34	± ± ± ± ± ±	1.7 4.5 3.52 8.22 0.53 0.13
W_max_ (W)	323	±	54
V̇O_2max_ (ml·min^−1^·kg^−1^)	49.3	±	3.9
HR_max_ (bpm)	192	±	8
RPE_max_ (6–20 points)	19.8	±	0.4
Blood lactate concentration at end of test (mmol·l^−1^)	10.41	±	1.50

Data are reported as mean ± SD. BMI, body mass index; *W*_max_, maximal workload; V̇O_2max_, maximal oxygen uptake; HR_max_, maximal heartrate; RPE, rate of perceived exertion.

### Study Design

After a maximal incremental cycling test on visit 1, participants performed two individually standardized, identical incremental cycling tests in a repeated measures non-randomized design without antioxidative supplementation (control, visit 2) and with 1 week of prior antioxidative supplementation (supplementation, visit 3), the two visits being at least 1 week apart.

#### Visit 1, Familiarization and Assessment of Maximal Workload

On the day of the visit 1, participants reported to the laboratory where they were familiarized with the testing procedures and the equipment, underwent assessment of body composition using dual-energy X-ray absorptiometry (lunar iDXA densitometer; GE Healthcare, Madison, WI, United States), and performed a maximal incremental cycling test. Equipped with a heart rate monitor (Polar M430; Polar Electro Inc., Kempele, Finland), an infrared sensor (Cosmed CPET, Rome, Italy) on their forehead, and a face mask, participants sat on an electromagnetically braked bicycle ergometer (Ergoline, Blitz, Germany) for 3 min. Then, they started cycling at 100 W with 30 W increments every 2 min until exhaustion. During exercise, gas exchange was measured breath by breath *via* a metabolic cart with integrated pulse oximetry (Cosmed CPET, Rome, Italy). At rest, every 2 min during exercise and at the point of exhaustion, a drop of capillary blood was taken for enzymatic analysis of blood lactate concentration (Biosen 5040-lactate analyzer; EKS Industrie Elektronik, Magdeburg, Germany). Additionally, 30 s before the end of every stage and at exhaustion, participants were asked to rank their rate of perceived exertion (RPE) on a Borg Scale ranging from 6 to 20 (Borg, 1982).

#### Visits 2 and 3, Experimental Trials

After a minimum of 6 days following visit 1, participants returned to the laboratory for visit 2 at 08:15 AM. After assessment of compliance with study restrictions, participants performed the first of two identical incremental cycling tests. The protocol consisted of a standardized incremental test starting at 20% of their individual maximal workload (W_max_; assessed on visit 1) and increased every 2 min by 20% W_max_ until 100% W_max_, resulting in 10 min of total cycling. Assessments were identical to those on visit 1.

Then, participants were given seven daily doses of Vitamin C (2 × 500 mg; Burgerstein Vitamine, Antistress AG, Rapperswil-Jona, Switzerland) and of Vitamin E (400 I.E, Burgerstein Vitamine) for intake between visits 2 and 3 which were scheduled 7 days apart. Participants were advised to take 500 mg vitamin C and 400 I.U. vitamin E every morning after breakfast and 500 mg vitamin C in the evening before going to bed. On the last day of supplementation, which was the very morning on the day of visit 3, participants were asked to take the entire daily dose of vitamins C and E after breakfast, which was scheduled approximately 2 h before reporting to the laboratory. The testing procedures and assessments were identical to visit 2.

### Blood Withdrawal and Processing

Ten minutes before (baseline) and at several timepoints after (0, 30, 90, 180, and 270 min post-exercise) the standardized incremental tests on visits 2 and 3, venous blood was drawn from an antecubital vein into 6 ml BD vacutainer tubes spray-coated with EDTA anti-coagulant for MNC isolation and 5 ml BD vacutainer rapid serum tubes with thrombin and a gel barrier (Becton Dickinson AG, Allschwil, Switzerland) for serum analysis. Throughout the duration of blood collection, participants remained in the laboratory conducting light office work or reading. Until completion of the last blood withdrawal, they were not allowed to shower, eat, or drink except for a standardized meal provided 2 h after exercise cessation and water which participants had access to *ad libitum* (consumed amount was reported in a log file).

All blood samples were processed within 3.5 h from timepoint of withdrawal. Serum was isolated *via* centrifugation and stored at −80°C until further analysis of TOC and TAC capacities. Human peripheral blood MNCs were isolated *via* density gradient centrifugation using Ficoll Histopaque (Ficoll-Paque™ Plus; GE Healthcare, Opfikon, Switzerland). Differential leukocyte counts were obtained in duplicate by a hematology analyzer (Beckman Coulter AC. T diff; Beckman Coulter, Fullerton, United States; intra-sample coefficient of variation for MNC counts = 3.05%, *n* = 23).

### Flow Cytometry

**Figure 1:**
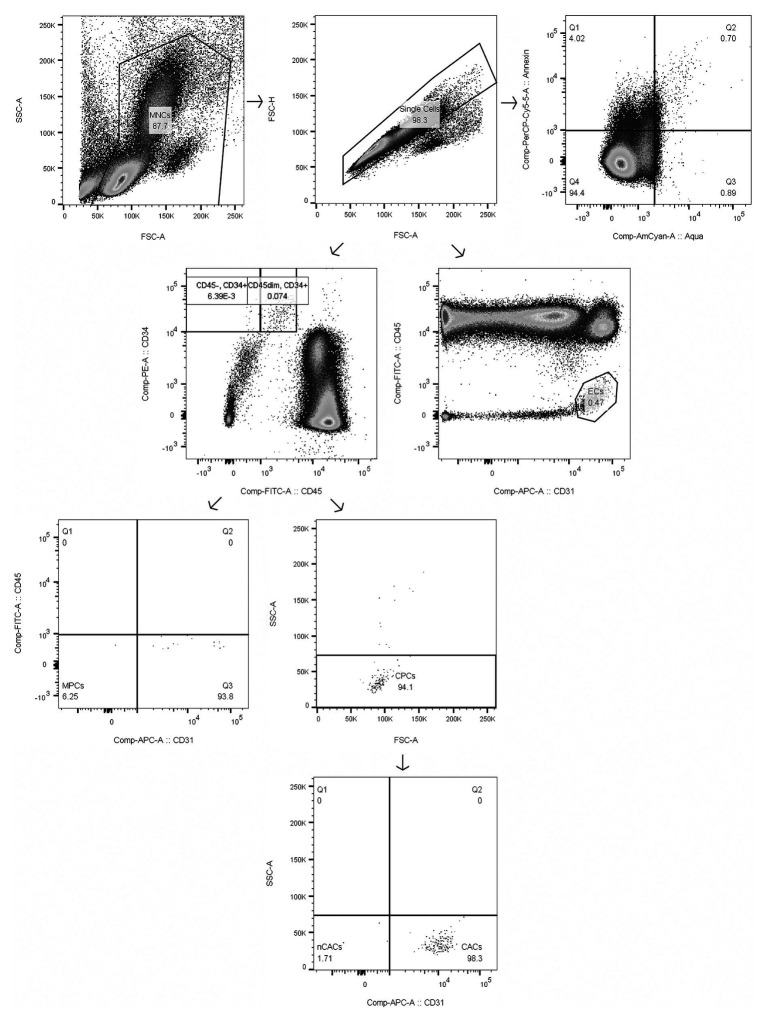
Gating strategy of mononuclear cells (MNCs), circulating angiogenic precursor cells (CACs), circulating non-angiogenic precursor cells (nCACs), mesenchymal precursor cells (MPCs), endothelial cells (ECs), and apoptotic MNCs as representative example of gating of apoptotic subsets. Numbers in boxes represent % of parent population.

For each timepoint of blood withdrawal, 1.5 × 10^6^ isolated human peripheral blood MNCs were labeled with the following fluorochrome-conjugated monoclonal antibodies in saturated concentrations: CD34-PE (Life Technologies Europe BV, Zug, Switzerland), CD45-FITC (Becton Dickinson AG, Allschwil, Switzerland), and CD31-APC (BioLegend Europe, London, United Kingdom) according to the manufacturer’s instruction. Cells were additionally stained with Aqua Zombie Dye (1:1,000; Life Technologies Europe BV, Zug, Switzerland) and Annexin V-PerCP-Cy5.5 (1:200; Becton Dickinson AG, Allschwil, Switzerland) for analysis of viability and apoptosis, respectively, according to the manufacturer’s instruction. Flow cytometry analysis was performed on a BD LSRII Fortessa (Becton Dickinson AG, Allschwil, Switzerland) – equipment of the flow cytometry facility, University of Zurich. 250,000 events in the single cell MNC gate were acquired and further analyzed using the FlowJo software (Treestar, Inc., San Carlos, United States). All samples were processed and analyzed in duplicates. For each participant, a fluorescence-minus-one sample for each antibody was analyzed as a control for correct gating, and compensation was performed to correct for spectral overlap. Cell subsets were defined as follows: CACs (CD34^+^/CD45^dim^/SSC^low^/CD31^+^), nCACs (CD34^+^/CD45^dim^/SSC^low^/CD31^−^), MPCs (CD34^+^/CD45^−^/CD31^−^), and ECs (CD31^+^/CD45^−^) – please find a representative example of our gating strategy in Figure 1.

Despite CD31 being considered a marker for cells of the endothelial lineage (Muller et al., 1989), more recent studies have shown that angiogenic hematopoietic stem cells also express CD31 (Duda et al., 2007; Vink et al., 2020). Cells lacking CD31 expression can be considered as non-angiogenic (Landers-Ramos et al., 2019).

Absolute estimates of cells/ml blood were obtained *via* multiplication of combined monocyte and lymphocyte counts from the hematology analyzer with the corresponding percentage values obtained during flow cytometry analysis. Cells were considered to be apoptotic when they stained positively for Annexin V – early apoptotic (Annexin V^+^/Aqua^−^) and late apoptotic (Annexin V^+^/Aqua^+^). Lymphocyte to monocyte ratios (LYM/MONO) resulting from both the hemocytometer analysis of the whole blood samples and the flow cytometry analysis of the processed MNC samples were compared, as it has previously been reported that unaccounted changes due to sample preparation might confound results if reporting live cells/ml after density gradient centrifugation (Kröpfl et al., 2019). However, due to the nature of our gating strategy where all cells were pre-gated on total single MNCs (FSC-A vs. FSC-H and FSC-A vs. SSC-A; Finak et al., 2016), a correction was not necessary as LYM/MONO changes from whole blood to flow cytometry analysis were negligible. Coefficient of variation for CACs (%MNCs) equaled 7.99%, *n* = 20.

### Total Oxidative Capacity and Total Antioxidative Capacity

Total oxidative capacity and total antioxidative capacity were analyzed in duplicates (Clinical Institute of Medical and Chemical Laboratory diagnostic, Medical University of Graz, Austria) by measuring the reaction of peroxides with peroxidase in serum using the corresponding TOC and TAC kits (DMP 20-4200T-O-C and DMP 20-4100T-A-C, Omnignostica GmbH., Höflein/Klosterneuburg, Austria) according to the manufacturer’s instructions. TOC values are given in equivalents of H_2_O_2_ (mmol/l), and TAC values as equivalents of Trolox (mmol/l). The oxidative stress index (TOC/TAC) is an indicator of any shift in redox balance (Harma and Erel, 2003; Bolukbas et al., 2005).

### Data Analysis and Statistics

Maximal oxygen uptake (V̇O_2max_) was defined as the highest moving time weighted average of all breaths within 30 s over the whole duration of the incremental test to exhaustion on visit 1. *W*_max_ was defined as the performance of the last completed stage [W] + the time cycled in the last stage(s)·120 s^−1^·30 W.

Two-way analyses of variance with a repeated-measures design were performed to investigate main effects of time and intervention, as well as interaction (time × intervention) for the different outcome parameters (CACs, nCACs, MPCs, ECs, MNCs, and their respective apoptotic subsets, as well as TOC and TAC). When significant differences were detected, *post hoc* analyses in form of Dunnet’s multiple comparisons test for interaction effects and main time effects (comparison of each timepoint to pre) were performed. For interaction effects, a multiple comparisons test with Sidak’s correction was additionally performed. Sphericity was tested with Mauchly’s test of sphericity. Wherever assumption of sphericity was violated, data were corrected by Greenhouse-Geisser.

Two-tailed Pearson correlations were performed to assess linear relations between two parameters at a given timepoint. Repeated measures correlations over multiple timepoints were calculated using the R package “rmcorr” provided by Bakdash and Marusich (2017) and are reported in the text by the coefficient *r*_rm_. Repeated measures correlations respect the assumption of independence of observations and yield greater statistical power compared to simple correlations.

Paired Student’s *t*-tests were performed to assess differences between visits 2 and 3 in all other variables such as percentage of V̇O_2max_ reached or maximal blood lactate concentrations. Whenever data were not normally distributed, Wilcoxon matched-pairs signed rank test was used.

Data are presented as arithmetic mean ± SD. Significance was set at *p* < 0.05. All statistical analyses were conducted using GraphPad Prism 8 for Mac (GraphPad Software Inc., San Diego, California Unites States), except for Mauchly’s test of sphericity which was performed using IBM SPSS Software version 25 (IBM Corp., Armonk, NY, United States), and repeated measures correlations which were calculated in R version 3.6.1 (R Core Team, 2019; R: A language and environment for statistical computing. R Foundation for Statistical Computing, Vienna, Austria).

## Results

All participants successfully completed all three visits. No adverse events were reported. Exercise physiological parameters from the control trial on visit 2 and the supplementation trial on visit 3 did not significantly differ (*p* > 0.05, Table 2). Exact time of test start was 08:40 AM ±9 min on visit 2 and 08:36 AM ±4 min on visit 3. Deviations of the actual timepoints of blood withdrawal from scheduled timepoints were never more than ±4 min.

**Table 2:** Exercise physiological parameters at the end of the standardized incremental cycling test (*n* = 18).

	Control trial (visit 2)	Supplementation trial (visit 3)	*p*
V̇O_2end_ (%V̇O_2max_)	95.5 ± 6.1	96.6 ± 8.0	0.437
HR_end_ (bpm)	182 ± 7	183 ± 9	0.643
RPE_end_ (6–20 points)	18.4 ± 1.0	18.6 ± 0.9	0.750
Blood lactate concentration (mmol·l^−1^)	7.60 ± 1.55	7.69 ± 1.63	0.758

Data are reported as mean ± SD. HR, heart rate; V̇O2, oxygen uptake; V̇O_2max_, maximal oxygen uptake; RPE, rate of perceived exertion.

### Total Oxidative Capacity and Total Antioxidative Capacity

**Figure 2:**
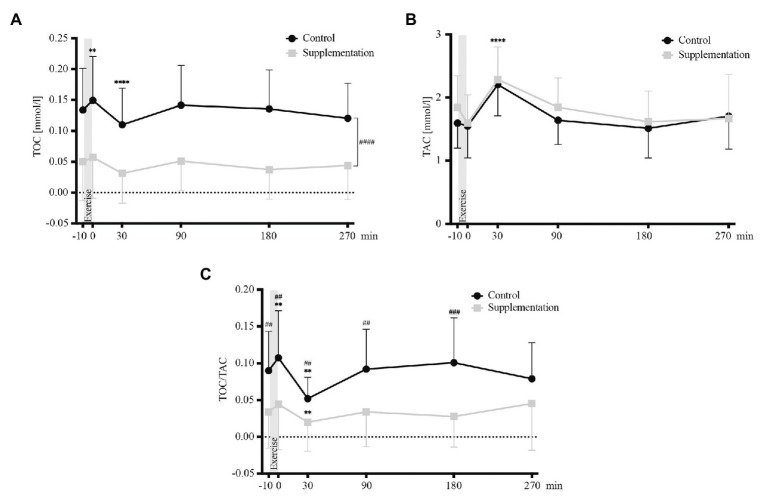
Total oxidative capacity (TOC) values **(A)**, TAC values **(B)**, and TOC/TAC ratio (**C**; *n* = 18). Values are mean ± SD. Differences from baseline are indicated by ^*^, with ^**^p < 0.01, ^****^p < 0.0001. Differences between the two interventions are indicated by ^#^, with ^##^*p* < 0.01, ^###^*p* < 0.001.

The absolute changes in TOC, TAC, and TOC/TAC ratio are depicted in Figure 2.

Analysis of variance revealed a significant main effect of intervention (control vs. supplementation) for TOC [*F*(1, 17) = 50.52, *p* = 0.0001], but not for TAC [*F*(1, 17) = 1.78, *p* = 0.2]. Furthermore, the analysis showed a significant effect of time (baseline vs. timepoints post-exercise) for both, TOC and TAC values [*F*(2.58, 43.78) = 9.82, *p* < 0.0001 and *F*(5, 85) = 20.16, *p* < 0.0001]. Follow-up tests showed that TOC values increased significantly directly after exercise (*p* = 0.003, Figure 2A) and that values 30 min after exercise cessation differed significantly from baseline for both parameters, with TOC values being significantly decreased (*p* < 0.0001, Figure 2A), while TAC values were increased (*p* < 0.0001, Figure 2B). However, no interaction effect was found for TOC or TAC [*F*(2.824, 48.01) = 0.55, *p* = 0.64 and *F*(5, 85) = 1.1, *p* = 0.37].

Additionally, there was a significant main effect of time [*F*(2.569, 43.67) = 7.4, *p* = 0.0007], intervention [*F*(1, 17) = 25.68, *p* < 0.0001], and interaction [*F*(2.505, 42.58) = 3.51, *p* = 0.03] for TOC/TAC. In detail, TOC/TAC increased significantly from baseline to directly post-exercise in the control trial (*p* = 0.009) and decreased significantly below baseline in both interventions (*p* = 0.001 for the control, *p* = 0.002 for the supplementation trial, respectively; Figure 2C). Significant differences between the two interventions existed at baseline (*p* = 0.004), directly after (*p* = 0.009), as well as 30 min (*p* = 0.004), 90 min (*p* = 0.002), and 180 min (*p* = 0.0001) after exercise (Figure 2C).

### Mononuclear Cells

**Figure 3:**
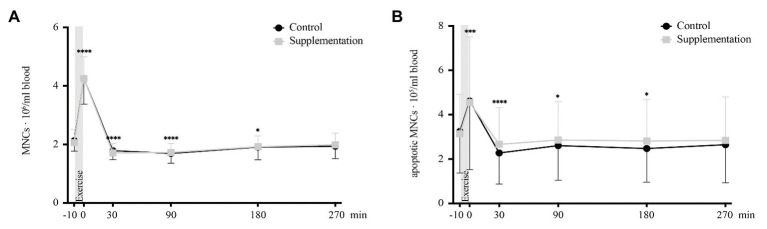
Total number of MNCs **(A)** and number of apoptotic MNCs **(B)** in cells/ml blood (*n* = 18). Values are mean ± SD. Differences from baseline are indicated by ^*^, with ^*^p < 0.05, ^***^p < 0.001, and ^****^p < 0.0001.

For total numbers of MNCs, ANOVA revealed a significant main effect of time [*F*(1.862, 31.66) = 209.9, *p* < 0.0001] but no intervention [*F*(1, 17) = 0.02, *p* = 0.89] or interaction [*F*(2.449, 41.63) = 0.39, *p* = 0.72] effects could be observed. Follow-up analysis showed a significant increase in MNC numbers from baseline to directly after exercise (*p* < 0.0001, Figure 3A) and a subsequent decrease below baseline 30, 90, and 180 min after exercise (*p* < 0.0001, *p* < 0.0001, and *p* = 0.036, respectively; Figure 3A).

Similar changes were seen for numbers of apoptotic MNCs, with a significant time effect [*F*(1.8312, 31.13) = 19.22, *p* < 0.0001] but again no main effect of intervention [*F*(1, 17) = 0.02, *p* = 0.67] or interaction effect [*F*(2.697, 45.84) = 0.78, *p* = 0.5]. *Post hoc* multiple comparisons revealed an increase in apoptotic MNC numbers directly after exercise (*p* = 0.0001, Figure 3B) with decreased numbers 30, 90, and 180 min post-exercise (*p* < 0.0001, *p* = 0.012, *p* = 0.015; Figure 3B) compared to baseline.

### Circulating Angiogenic Precursor Cells

**Figure 4:**
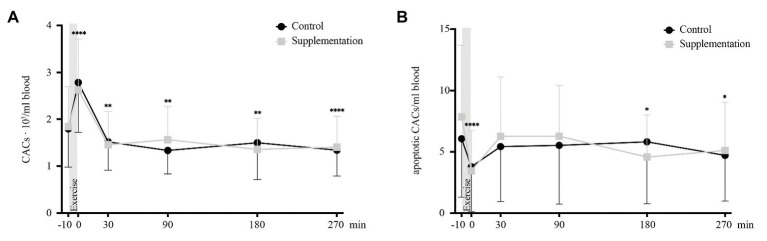
Total number of CACs **(A)** and number of apoptotic CACs **(B)** in cells/ml blood (*n* = 18). Values are mean ± SD. Differences from baseline are indicated by ^*^, with ^*^p < 0.05, ^**^p < 0.01, and ^****^p < 0.0001.

A significant effect of time was found for total numbers of CACs [*F*(2.051, 34.86) = 47.64, *p* < 0.0001]. However, no intervention [*F*(1, 17) = 0.001, *p* = 0.98] or interaction [*F*(5, 85) = 1.63, *p* = 0.16] effects were observed. Comparable to MNCs, total CACs increased directly after exercise (*p* < 0.0001) and dropped below baseline from 30 min after (*p* = 0.001) until the last timepoint of blood withdrawal (90 min after *p* = 0.002, 180 min after *p* = 0.002, and 270 min after *p* < 0.0001; Figure 4A).

The apoptotic subset of CACs also showed a significant main effect of time [*F*(5, 85) = 5.40, *p* = 0.0002], no difference between the two interventions [*F*(1, 17) = 0.2, *p* = 0.66], and no interaction effect [*F*(5, 85) = 1.47, *p* = 0.21]. When compared to baseline concentrations, apoptotic CACs were significantly lower directly post-exercise (*p* < 0.0001), as well as 180 and 270 min after cycling (*p* = 0.049 and *p* = 0.016, Figure 4B).

### Circulating Non-angiogenic Precursor Cells

**Figure 5:**
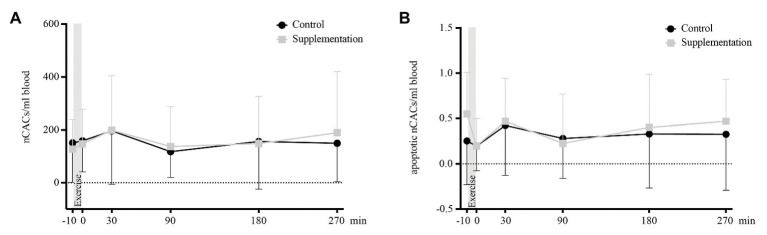
Total number of nCACs **(A)** and number of apoptotic nCACs **(B)** in cells/ml blood (*n* = 18). Values are mean ± SD.

For total and apoptotic nCAC numbers, neither the main effects of time [*F*(2.444, 41.54) = 2.33, *p* = 0.10 and *F*(5, 85) = 2.19, *p* = 0.06] or intervention [*F*(1, 17) = 0.01, *p* = 0.91 and *F*(1, 17) = 0.67, *p* = 0.43], or interaction effects [*F*(5, 85) = 1.12, *p* = 0.36 and *F*(5, 85) = 0.74, *p* = 0.59] were of statistical significance. A graphic display of nCAC kinetics is given in Figure 5.

### Mesenchymal Precursor Cells

**Figure 6:**
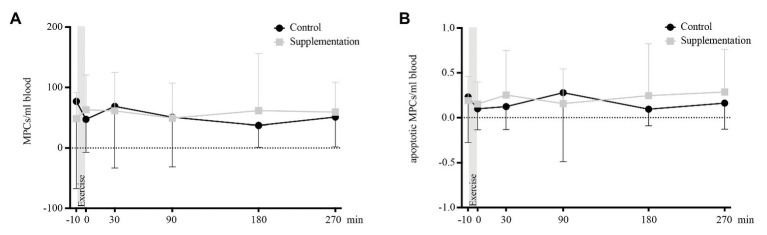
Total number of MPCs **(A)** and number of apoptotic MPCs **(B)** in cells/ml blood (*n* = 18). Values are mean ± SD.

There were neither any main effects of time or intervention, nor an interaction effect on total [*F*(2.866, 48.73) = 0.68, *p* = 0.57, *F*(1, 17) = 0.02, *p* = 0.9, and *F*(1.769, 30.08) = 1.15, *p* = 0.33] or apoptotic MPCs [*F*(5, 85) = 0.32, *p* = 0.9, *F*(1, 17) = 0.56, *p* = 0.47, and *F*(5, 85) = 0.68, *p* = 0.64]. Kinetics of MPCs is depicted in Figure 6.

### Mature Endothelial Cells

**Figure 7:**
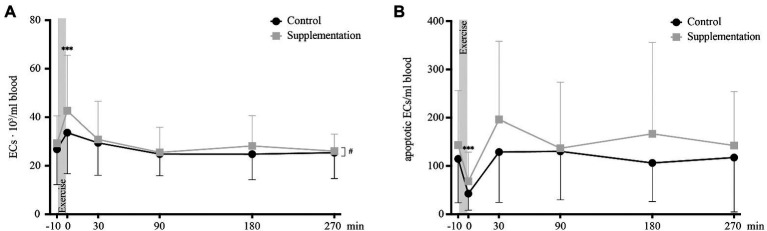
Total number of ECs **(A)** and number of apoptotic ECs **(B)** in cells/ml blood (*n* = 18). Values are mean ± SD. Differences from baseline are indicated by ^*^, with ^***^p < 0.001. Differences between the two interventions are indicated by ^#^, with ^#^*p* < 0.05.

Overall numbers of circulating ECs showed a significant main effect of time [*F*(2.303, 39.16) = 8.55, *p* = 0.0005], as well as a main effect of intervention [*F*(1, 17) = 5.3, *p* = 0.034]. However, there was no interaction effect [*F*(3.289, 55.92) = 1.85, *p* = 0.14]. Follow-up analysis showed that the only significant change occurred directly after exercise where EC numbers were increased compared to baseline values (*p* = 0.0009, Figure 7A).

For apoptotic EC numbers, the only effect observed was over time [*F*(5, 85) = 8.29, *p* < 0.0001], while no intervention or interaction effects could be found [*F*(1, 17) = 4.06, *p* = 0.06 and *F*(2.396, 40.730) = 0.72, *p* = 0.15, respectively]. Specifically, a change in apoptotic ECs was seen from baseline to 0 min after, where apoptotic EC numbers significantly decrease (*p* = 0.0004, Figure 7B).

### Correlations

A Pearson correlation analysis between TOC/TAC and apoptotic MNCs directly after exercise in the control intervention was not significant [*r*(16) = 0.03, *p* = 0.92]. Repeated measures correlations between TOC/TAC and CACs, nCACs, and MPCs over all timepoints revealed neither significances for the control [CACs: *r*_rm_(89) = 0.13, *p* = 0.21; nCACs: *r*_rm_(89) = −0.09, *p* = 0.38; and MPCs: *r*_rm_(89) = 0.04, *p* = 0.68] nor the supplementation intervention [CACs: *r*_rm_(89) = 0.09, *p* = 0.42; nCACs: *r*_rm_(89) = −0.03, *p* = 0.8; and MPCs: *r*(89) = −0.14, *p* = 0.19].

Repeated measures correlations between apoptotic MNCs and CACs showed a significant correlation for both, the control [*r*_rm_(89) = 0.73, *p* < 0.0001], as well as for the supplementation [*r*_rm_(89) = 0.53, *p* < 0.0001] intervention. However, a Pearson correlation between numbers of apoptotic MNCs and the increase in CACs directly after exercise was not significant [*r*(16) = 0.41, *p* = 0.09 for the control and *r*(16) = −0.05, *p* = 0.42 for the supplementation intervention, respectively].

Further, the significantly increased number of CACs directly after exercise did also not correlate with TOC/TAC [*r*(16) = −0.04, *p* = 0.88 for the control and *r*(16) = 0.05, *p* = 0.85 for the supplementation intervention, respectively], TOC [*r*(16) = −0.07, *p* = 0.78 for the control and *r*(16) = 0.28, *p* = 0.27 for the supplementation intervention, respectively], or TAC concentrations [*r*(16) = 0.05, *p* = 0.86 for the control and *r*(16) = 0.12, *p* = 0.64 for the supplementation intervention, respectively] directly after exercise.

## Discussion

The present study investigated the effects of exercise-induced oxidative stress on circulating numbers of CACs, nCACs, MPCs, ECs, and their apoptotic subsets after an acute bout of incremental cycling. A special focus was laid on numbers of circulating apoptotic MNCs as potential mediator of direct or delayed precursor cell mobilization.

We found antioxidative supplementation to abolish the observed exercise-induced increase in TOC/TAC and lower oxidative stress levels at baseline and up to 180 min after exercise. Further, we found significant increases in CACs and apoptotic MNCs directly after exercise which were not affected by supplementation. Numbers of nCACs and MPCs did not show any significant changes.

Interestingly, an intervention and time effect but no interaction effect was observed for TOC. The supplementation lowered TOC concentrations *per se* without affecting its exercise-induced kinetics. TOC values increased directly post-exercise, regardless of the supplementation status. Moreover, a subsequent decrease in TOC below baseline was seen 30 min post-exercise under both conditions. This decrease could be explained by the simultaneous increase in TAC. In contrast to TOC, however, TAC was not affected by the exogenous antioxidant supplementation. This suggests that overall TAC concentrations, as well as the observed increase 30 min after exercise, are predominantly determined by the activity of the body’s inherent antioxidative system. It could counteract the effect of antioxidant supplementation by downregulating the activity or concentration of endogenous enzymes such as superoxide dismutase or glutathione peroxidase under resting conditions (Duthie et al., 1990). Acute exercise could induce an upregulation of these enzymes (Dekany et al., 2006). The hypothesis of exercise stimulating the endogenous antioxidative system is in agreement with findings of trained athletes showing higher TAC values compared to sedentary controls. A chronic series of repeated exercise stimuli had a positive impact on the potency of their endogenous antioxidative system (Criswell et al., 1993). One could now speculate, whether supplementation of exogenous antioxidants such as vitamin C and E over longer time periods could even lower the potency of the body’s inherent antioxidative system, as already discussed in athletes (Nocella et al., 2019).

Given the disparate effects of exercise and antioxidative supplementation on TOC and TAC, it is important to keep in mind that the physiology of the redox system is highly dynamic and that oxidative stress by definition represents a shift toward the oxidative side of the system (Sies et al., 2017). Therefore, reporting the TOC/TAC ratio presents a more accurate picture of the true change in oxidative stress (Harma and Erel, 2003; Kosecik et al., 2005; Karababa et al., 2013). Baseline TOC/TAC levels were reduced and the increase directly after exercise was no longer significant upon supplementation, which is in line with our first hypothesis.

Interestingly, findings of this aforementioned TOC/TAC reduction did not translate into reduced numbers of apoptotic MNCs – neither at baseline, nor directly after exercise. There was no significant correlation between TOC/TAC and apoptotic MNCs directly after exercise, which suggests that oxidative stress was not the main driver of MNC apoptosis during exercise. As a consequence, we reject our second hypothesis.

Our findings of an immediate but transient increase in numbers of MNCs after exercise agree with already published results (Bonsignore et al., 2002; Moebius-Winkler et al., 2009; Baker et al., 2017). However, we extend existing literature by showing that the apoptotic MNC subset has similar kinetics. Numbers increased directly after exercise cessation, but then dropped below baseline 30 min and stayed there up to 180 min post-exercise. This observation speaks against the theory of undershoot of mature immune cell numbers post-exercise (lymphocytopenia) being due to increased cell apoptosis, which has already been controversially discussed (Mooren et al., 2002; Simpson et al., 2007). In addition, it further corroborates the dismantling of the “open window”-theory of an impaired immune system after exercise by Campbell and Turner, where the authors argue that observed reductions in lymphocyte numbers and function after exercise reflect a transient and time-dependent redistribution of immune cells to peripheral tissues, even resulting in a heightened state of immune surveillance and regulation rather than immune suppression (Campbell and Turner, 2018). Our results are in line with this thought by showing that the amount of apoptotic cells among overall MNCs – reflecting the degree of immune suppression – dropped below baseline 30–180 min after the intense acute exercise.

Our third hypothesis was that apoptotic MNCs correlate with the amount of mobilized precursor cells – either directly or with a delayed effect. This hypothesis is based on a study by Mooren and Krüger reporting increased HPC (defined as Sca-1^+^/c-kit^+^) and EPC (defined as Sca-1^+^/Flk^+^) numbers 3 h after injection of apoptotic CD3^+^ lymphocytes in a mouse model (Mooren and Krüger, 2015). Our human data, however, does not support these results, since neither CACs, nCACs nor MPCs showed any delayed increase. The only significant elevation occurred for CACs directly after exercise and was simultaneous to an increase in apoptotic MNCs. And even though the number of apoptotic MNCs significantly correlated with CACs across all timepoints, numbers directly after exercise did not. This indicated that the immediate post-exercise increase in CACs was not mediated by the immediate increase in apoptotic MNCs. However, the link between apoptotic MNCs and CACs over all timepoints could possibly be explained by a mechanism not only specific to acute exercise. In the context of cell death by apoptosis, small vesicles are formed that carry significant amounts of biologically active molecules such as protein, lipids, messenger, or micro RNAs that exert beneficial regenerative effects (Beer et al., 2016). These compounds are secreted and could stimulate a common stem cell recruiting gradient such as the SDF-1/CXCR-4 axis (Cheng et al., 2019). Thus, a potential role of apoptotic MNCs in the regulation of precursor cell mobilization after exercise warrants further investigation, especially because SDF-1 was found to be elevated after acute exercise (Niemiro et al., 2017).

Comparing our findings of an increase in CAC numbers directly after an acute bout of exercise with existing literature, we find an agreement with previously published reports of CAC (Shill et al., 2016; O’Carroll et al., 2019), EPC (Bonsignore et al., 2010; Krüger et al., 2015; Montgomery et al., 2019), and HPC kinetics (Krüger et al., 2015; Baker et al., 2017). However, it is apparent that their definitions vary and especially for HPCs CD31-expression is usually not taken into account; Krüger et al. (2015) found acute exercise to increase HPCs defined as CD34^+^/CD45^+^, while Baker et al. (2017) defined them only as CD34^+^ and also found numbers to be increased after exercising at a high but not at a moderate intensity. Another study defined HPCs as CD34^+^/CD45^low^ and found them to be elevated during but not directly after an acute exercise bout (Niemiro et al., 2017). Our results showed that only CD34^+^/CD45^dim^/SSC^low^/CD31^+^ cell numbers were elevated after acute exhaustive exercise. This adds to the existing knowledge about exercise-induced HPCs that their increase is mostly mediated by cells with angiogenic potential, while numbers of nCACs did not change after the acute exercise.

A more inconsistent picture presents itself when looking at the MPC data, where our observations contradict Ramírez et al. (2006) finding of MPCs (defined as medium-high forward and side scatter, CD4^−^/CD2^+^/CD13^+^ events) being increased post-exercise, but agree with the results of Niemiro and colleagues where MPCs (CD45^−^/CD31^−^/CD105^+^) did not change after exercise cessation (Niemiro et al., 2017). A possible explanation for these diverging results could lie in the type of exercise that was conducted. The first study by Ramírez et al. (2006) measured MPCs after a long-distance run (21 km) which induced acute skeletal muscle injury, likely resulting in the activation and mobilization of MPCs for regenerative purposes. In the second study by Niemiro et al. (2017) participants completed 60 min of running on a treadmill at 70% V̇O_2max_ – a similar exercise stimulus as used in this study. The acute exercise did not cause enough skeletal muscle injury to result in any mobilization of MPCs from the bone marrow niche.

Baseline and exercise-induced precursor cell numbers highly depend on their underlying definition criteria. Literature findings have to be compared and evaluated with great caution, even though commonly employed nomenclature of cell subgroups seems to suggest uniformity.

Therefore, a strength of the present study is the identification of precursor cell subgroups by the application of stringent definition criteria using a simple antibody panel that only targets three clusters of differentiation (CD34, CD45, and CD31). They represent three of the most commonly reported precursor cell markers in literature. This methodological approach facilitates data comparison with future original research and proposes a simple and reproducible way of dividing a population of general CD34^+^-stem cells into three non-overlapping subgroups of precursor cells, which we call CACs, nCACs, and MPCs. We are, however, fully aware that the described subgroups are not exclusive and might contain heterogenous cell groups that differ in lineage-specificity or developmental status, as precursor cell differentiation is a continuous and highly dynamic process.

To our best knowledge, the present study is the first to not only investigate the kinetics of CACs, nCACs, MPCs, ECs, and MNCs but also their apoptotic subsets in response to exercise until 270 min post-exercise in humans. We found the aforementioned acute increase in CACs to be accompanied by a concomitant decrease in apoptotic CACs, meaning that acute exercise not only increased the overall quantity but also the percentage of truly viable CACs. This suggests that the observed increase was most likely due to an acute mobilization of CACs (i.e., EPCs and angiogenic HPCs) from their niches, which for EPCs – as recently reported – is thought to be located within the vessel wall rather than the bone marrow (Fujisawa et al., 2019). However, increased cell viability might not necessarily translate into an increased proliferative capacity and colony growth (Rummens et al., 2012; Stelzer et al., 2015; Witkowski et al., 2016).

Similar to their immature counterparts, numbers of mature circulating ECs significantly increased directly after exercise. We suggest this EC elevation is predominantly caused by endothelial shedding due to increased laminar shear stress during exhausting physical activity (Moebius-Winkler et al., 2009). However, other exercise-induced stressors, such as heat production, inflammation, or oxidative stress might also have played a role for the release of mature ECs into the blood stream (Marsh and Coombes, 2005). Interestingly, numbers of circulating ECs return back to baseline already 30 min post-exercise. This decrease is accompanied by an increase in apoptotic circulating ECs back to baseline levels, indicating mature ECs that are shed off the vessel wall during exercise quickly commit apoptosis when circulating freely in the blood stream. The physical detachment of endothelial cells, for example, as a response to prolonged exposure to shear or oxidative stress, involves the loss of cell-cell contacts as well as contact to anchoring proteins and initiates pro-apoptotic signals (Rabelink et al., 2010). Even though circulating ECs represent a marker for endothelial damage (Erdbruegger et al., 2006), and thus their increase would indicate exercise to be harmful to the vasculature, the concomitant increase in CACs suggests a rapid restoration of the endothelial layer which is possibly even a necessary remodeling process in the long-term establishment of beneficial vascular adaptions to exercise training (Sapp and Hagberg, 2018).

In addition, we found average numbers of circulating ECs to be significantly different after supplementation. Whether this phenomenon underlies an actual effect of vitamin intake on EC quantity (i.e., endothelial shedding) and if so, whether this effect is mediated *via* a reduction in oxidative stress, warrants further investigation.

The lack of randomization of the order of the two interventions, a placebo control or a control accounting for intra-day variability is a limitation of the study. However, we chose to prioritize having similar individual and environmental conditions for each subject rather than randomizing and having tests several months apart due to the long washout period of vitamin E (Chuang et al., 2011). Additionally, no measurement of the cell internal redox status was performed and thus the herein reported oxidative index only mimics blood/serum conditions experienced by circulating cells. Furthermore, cell subgroups were named “precursor cells” instead of “stem and progenitor cells” in order to acknowledge the possibly non-exclusive definition of the different phenotypes by our antibody panel and gating strategy.

In conclusion, our findings suggest that a reduction in exercise-induced oxidative stress, induced by antioxidative supplementation, does not affect the transient increase in numbers of CACs or apoptotic MNCs directly after exercise cessation or with a delayed effect. Moreover, numbers of apoptotic MNCs did not correlate to CACs numbers directly post-exercise; however, the correlation over all timepoints between parameters was significant. Future investigation will clarify the underlying mechanism, which is possibly not only specific to acute exercise. Furthermore, neither the acute exercise nor the antioxidative supplementation altered numbers of CACs and MPCs.

## Data Availability Statement

The raw data supporting the conclusions of this article will be made available by the authors, without undue reservation.

## Ethics Statement

The studies involving human participants were reviewed and approved by Ethics Committee of the canton of Zurich (project ID: BASEC 2018-02075). The patients/participants provided their written informed consent to participate in this study.

## Author Contributions

CS, JK, and MS designed the study. MS collected the data. MS and JK analyzed the data. H-JG conducted the serum analysis. MS, JK, and CS discussed the data and wrote the manuscript. All authors read and approved the final manuscript.

### Conflict of Interest

The authors declare that the research was conducted in the absence of any commercial or financial relationships that could be construed as a potential conflict of interest.

## Abbreviations

BMI: Body mass index
CAC(s): Circulating angiogenic precursor cells
EC(s): (mature) endothelial cells
EPC(s): Endothelial precursor cells
HPC(s): Hematopoietic precursor cells
HR_max_: Maximal heartrate
MNC(s): Mononuclear cells
MPC(s): Mesenchymal precursor cells
nCAC(s): Circulating non-angiogenic precursor cells
RPE: Rate of perceived exertion
TAC: Total antioxidative capacity
TOC: Total oxidative capacity
V̇O_2max_: Maximal oxygen uptake
*W*_max_: Maximal workload
