# Corrigendum: Acute Exercise-Induced Oxidative Stress Does Not Affect Immediate or Delayed Precursor Cell Mobilization in Healthy Young Males

**DOI:** 10.3389/fphys.2021.775610

**Published:** 2021-10-14

**Authors:** Michelle Schmid, Hans-Jürgen Gruber, Julia M. Kröpfl, Christina M. Spengler

**Affiliations:** ^1^Exercise Physiology Lab, Institute of Human Movement Sciences and Sport, ETH Zurich, Zurich, Switzerland; ^2^Clinical Institute of Medical and Chemical Laboratory Diagnostics, Medical University of Graz, Graz, Austria; ^3^Zurich Center for Integrative Human Physiology (ZIHP), University of Zurich, Zurich, Switzerland

**Keywords:** stem cell mobilization, hematopoietic, endothelial, mesenchymal, apoptosis, total oxidative capacity, oxidative stress index, acute exhaustive exercise

In the published article, the significance was mistakenly placed at 30 min instead of 0 min in *Figure 7B* as published. The corrected *Figure 7* appears below.

**Figure 7:**
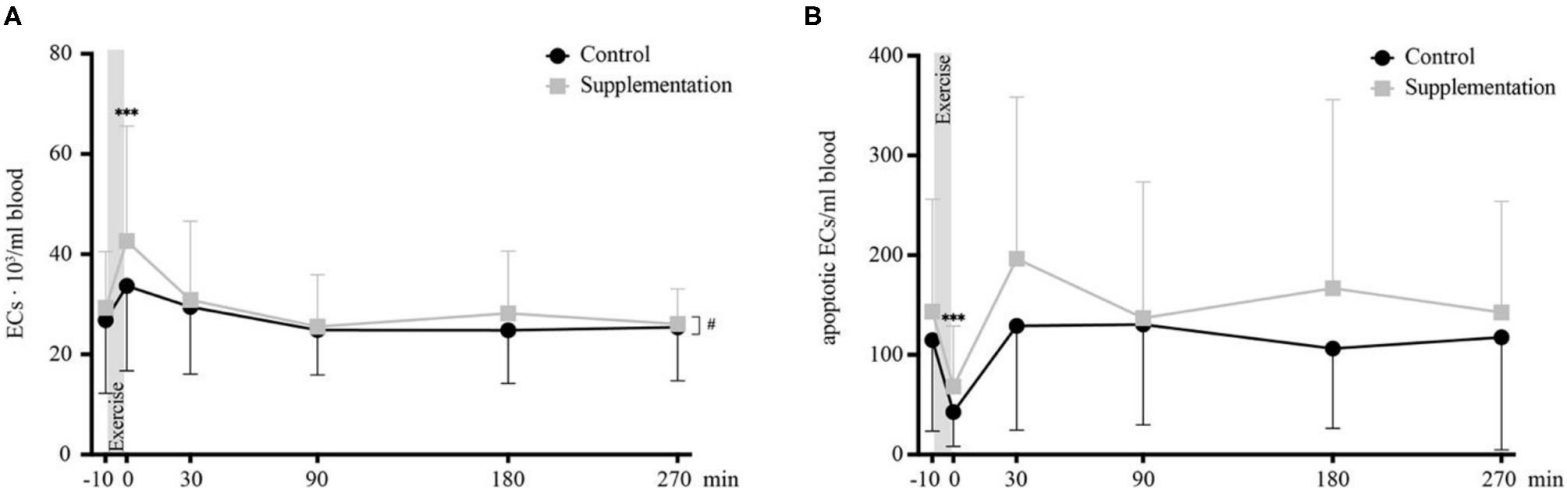
Total number of ECs **(A)** and number of apoptotic ECs **(B)** in cells/ml blood (*n* = 18). Values are mean ± SD. Differences from baseline are indicated by *, with ***p < 0.001. Differences between the two interventions are indicated by ^#^, with ^#^*p* < 0.05.

As a consequence, corrections have been made to:

**Results, Mature Endothelial Cells, Paragraph 2:**

“For apoptotic EC numbers, the only effect observed was over time [*F*_(5, 85)_ = 8.29, *p* < 0.0001], while no intervention or interaction effects could be found [*F*_(1, 17)_ = 4.06, *p* = 0.06 and *F*_(2.396, 40.730)_ = 0.72, *p* = 0.15, respectively]. Specifically, a change in apoptotic ECs was seen from baseline to 0 min after, where apoptotic EC numbers significantly **decrease** (*p* = 0.0004, **Figure 7B**).”

**Discussion, Paragraph 13**:

“This decrease is accompanied by an increase in apoptotic circulating ECs **back to baseline levels**, indicating mature ECs that are shed off the vessel wall during exercise quickly commit apoptosis when circulating freely in the blood stream.”

Please also note that although data were correctly displayed, the wording in one sentence in the Results section (**Results, Circulating Angiogenic Precursor Cells, Paragraph 2**) is misleading. It has to read: “When compared to baseline concentrations, apoptotic CACs were significantly lower directly post-exercise (*p* < 0.0001), as well as 180 and 270 min after cycling (*p* = 0.049 and *p* = 0.016, **Figure 4B**).”

The authors apologize for this error and state that this does not change the scientific conclusions of the article in any way. The original article has been updated.

## Publisher's Note

All claims expressed in this article are solely those of the authors and do not necessarily represent those of their affiliated organizations, or those of the publisher, the editors and the reviewers. Any product that may be evaluated in this article, or claim that may be made by its manufacturer, is not guaranteed or endorsed by the publisher.

